# A randomized, double-blind, placebo-controlled, Phase I/II trial of RNActive^®^-vaccine cv9104 in patients with metastatic castrate-refractory prostate cancer (mcrpc): first results of the Phase I part

**DOI:** 10.1186/2051-1426-2-S3-P85

**Published:** 2014-11-06

**Authors:** Sven D Koch, Henoch Hong, Susan Feyerabend, Margitta Retz, Hubert Kuebler, Axel Heidenreich, Thomas van Erps, Andreas Schroeder, Birgit Scheel, Valérie Reus, Karl-Josef Kallen, Mariola Fotin-Mleczek, Ulrike Gnad-Vogt, Arnulf Stenzl

**Affiliations:** 1 CureVac GmbH, Tübingen, Germany; 2 Study Practice Urology, Nuertingen, Germany; 3 Dept. of Urology, University Hospital, Technical University Munich, Germany; 4 Dept. of Urology, University Hospital, Technical University Aachen, Germany; 5 Curevac GmbH, Frankfurt, Germany; 6 CureVac GmbH, Germany; 7 Dept. of Urology, University Hospital, University of Tuebingen, Germany

## Introduction

CV9104 is a novel mRNA-based therapeutic vaccine for prostate cancer encoding the prostate cancer-associated antigens PSA, PSMA, PSCA, STEAP, PAP and MUC1 by engineered mRNA molecules optimized for protein expression and immunostimulation (RNActive^®^). CV9103, an mRNA-based vaccine against 4 of these antigens was previously shown to be safe and immunogenic in a pPhase I/IIA trial. Immune responses against multiple antigens were associated with improved survival in patients with mCRPC.

## Design and methods

Safety and immunogenicity of first human exposure to CV9104 was investigated in the Phase I part. In the Phase II part, chemotherapy-naïve patients with a- or minimally symptomatic mCRPC were randomized to CV9104 or placebo. Primary endpoint is overall survival. Secondary endpoints include progression free survival, quality of life and immunogenicity among others. Upon disease or symptom progression blinded treatment is continued in combination with the subsequent systemic cancer treatment until second progression. Blood samples for immune monitoring and other biomarkers were taken at baseline, week 6 (1 week post fourth vaccination) and week 24 (6 weeks post ninth vaccination). Cellular and humoral immune responses against all vaccine antigens were analyzed by intracellular cytokine staining and IgM and IgG ELISA, respectively. Blood leukocyte phenotyping was performed by flow cytometry. Transcriptome analyses in whole blood were performed and serum samples are currently analyzed for miRNAs.

## Results

Seven patients were enrolled in the Phase I part. Six of these patients were evaluable for immune responses. The independent data monitoring board recommended continuation into Phase II since nature and severity of adverse events were favorable and in line with previous results from CV9103 Recruitment of the Phase II part was finished in 12/2013 with 197 patients randomized. The trial is ongoing.

5 of 6 patients of the Phase I part exhibited antigen-specific immune responses post vaccination. All CV9104 antigens were immunogenic. Multiple immune responses against ≥2 antigens were observed in 2 patients. In addition, phenotypic changes in CD4+ and CD8+ T cells consistent with an effector phenotype were observed.

## Conclusion

Results from the Phase I part suggest that the self-adjuvanted mRNA vaccine CV9104 constitutes a well-tolerated approach to induce antigen-specific cellular and humoral immune responses against multiple antigens.

Trial registration: http://www.clinicaltrials.gov/ct2/show/NCT01817738.

